# Transcriptome analysis of embryonic mammary cells reveals insights into mammary lineage establishment

**DOI:** 10.1186/bcr2928

**Published:** 2011-08-11

**Authors:** Olivia Wansbury, Alan Mackay, Naoko Kogata, Costas Mitsopoulos, Howard Kendrick, Kathryn Davidson, Christiana Ruhrberg, Jorge S Reis-Filho, Matthew J Smalley, Marketa Zvelebil, Beatrice A Howard

**Affiliations:** 1 Breakthrough Breast Cancer Research Centre, The Institute of Cancer Research, 237 Fulham Road, London SW3 6JB, UK; 2 UCL Institute of Ophthalmology, University College London, Bath Street, London EC1V, UK

## Abstract

**Introduction:**

The mammary primordium forms during embryogenesis as a result of inductive interactions between its constitutive tissues, the mesenchyme and epithelium, and represents the earliest evidence of commitment to the mammary lineage. Previous studies of embryonic mouse mammary epithelium indicated that, by mid-gestation, these cells are determined to a mammary cell fate and that a stem cell population has been delimited. Mammary mesenchyme can induce mammary development from simple epithelium even across species and classes, and can partially restore features of differentiated tissue to mouse mammary tumours in co-culture experiments. Despite these exciting properties, the molecular identity of embryonic mammary cells remains to be fully characterised.

**Methods:**

Here, we define the transcriptome of the mammary primordium and the two distinct cellular compartments that comprise it, the mammary primordial bud epithelium and mammary mesenchyme. Pathway and network analysis was performed and comparisons of embryonic mammary gene expression profiles to those of both postnatal mouse and human mammary epithelial cell sub-populations and stroma were made.

**Results:**

Several of the genes we have detected in our embryonic mammary cell signatures were previously shown to regulate mammary cell fate and development, but we also identified a large number of novel candidates. Additionally, we determined genes that were expressed by both embryonic and postnatal mammary cells, which represent candidate regulators of mammary cell fate, differentiation and progenitor cell function that could signal from mammary lineage inception during embryogenesis through postnatal development. Comparison of embryonic mammary cell signatures with those of human breast cells identified potential regulators of mammary progenitor cell functions conserved across species.

**Conclusions:**

These results provide new insights into genetic regulatory mechanisms of mammary development, particularly identification of novel potential regulators of mammary fate and mesenchymal-epithelial cross-talk. Since cancers may represent diseases of mesenchymal-epithelial communications, we anticipate these results will provide foundations for further studies into the fundamental links between developmental, stem cell and breast cancer biology.

## Introduction

The mammary lineage is first established during organogenesis of the mammary primordium that forms during embryogenesis. Mammary primordia, comprised of mammary epithelial and associated mesenchymal tissues, develop as a result of inductive crosstalk between the surface epithelium and underlying mesenchyme [1]. During mouse embryogenesis, a multilayered epithelial mammary placode is evident at embryonic day (E) 11.0, which reaches the bud stage by E12.0. When E13.0 mammary primordial epithelium and salivary mesenchymal tissue are co-cultured, they produce a ductal tree that has morphological features of salivary ducts, but is capable of secreting milk [2]. These results suggest that by E13.0, mammary epithelium has been stably determined as mammary. Mammary mesenchyme can induce mammary differentiation in non-mammary epithelium, even from epithelium of other mammalian species and across classes, suggesting that the inductive signals provided by the mammary mesenchyme and the responding elements in the uninduced epithelium are highly conserved during mammalian evolution [1]. Together, the embryonic mammary epithelial and mesenchymal tissues act together as a signalling centre and communicate back and forth to each other to mediate all stages of embryonic mammary morphogenesis.

Another key aspect of organogenesis is the formation of tissue-specific stem cells. To identify at what developmental stage these stem cells first form, mammary primordia from different embryonic stages have been implanted into fat pads cleared of mammary epithelium to evaluate their repopulation capacity, which provides a measure of stem cell activity within a population. In these experiments, mammary primordia from E12.5 onwards formed extensive arborised ducts and have been used to evaluate postnatal mammary phenotypes in mice with genetic mutations which exhibit embryonic lethal phenotypes [3]. Moreover, mammary epithelium, isolated from E13.0 mammary primordia, independent of mammary mesenchyme, can give rise to all mammary epithelial cell types in this assay, and can even induce the formation of milk-secreting mammary alveoli in fat pads of pregnant females [4]. These observations suggest that tissue-specific stem cells reside within the primordial mammary epithelium by mid-gestation and that these cells have the ability to respond to the postnatal microenvironment in an appropriate fashion. However, the molecular identity of the epithelial cells in the mammary primordia is still ill defined, and the degree of similarity to postnatal mammary epithelial cells remains largely unknown.

To investigate new candidates for inductive tissue interactions in the mammary primordia, we have now profiled the gene expression signatures of purified mammary primordia and their epithelial and mesenchymal tissues in mid-gestation mouse embryos to identify genes specific for both cellular subpopulations. We have further compared the embryonic mammary expression patterns to their postnatal descendants using existing array data for the basal, luminal estrogen receptor positive and luminal estrogen receptor negative subpopulations of [5] to identify candidate markers for mammary progenitor regulators. Comparison of the mammary mesenchymal and postnatal stromal signatures provides new insights into likely effectors of mammary cell fate, lineage promotion and maintenance. Together, our results provide a global picture of signalling networks that may regulate key aspects of mammary organogenesis, including inductive signalling events, the initial establishment of the mammary lineage and mammary progenitor behaviour.

## Materials and methods

### Sample collection

All animal work was carried out under UK Home Office project and personal licenses following local ethical approval and in accordance with local and national guidelines. Embryonic day 12.5 (E12.5) mammary primordia number four were manually micro-dissected from FVB/N embryos in ice-cold D-PBS so that a thick layer of mesenchyme was left associated with the mammary epithelial bud. Based on histological and confocal data, the layer of mesenchymal cells consisted of a depth of approximately 8 to 16 cells and should include mostly estrogen receptor-alpha (ERα) expressing cells. Similar micro-dissected samples were digested for two minutes with pancreatin and trypsin, rinsed extensively in D-PBS to remove the enzymes. One hour later, primordia were separated with needles into two cell compartments, the mammary bud epithelium and the mammary bud-associated mesenchyme as described by [6]. All samples were collected in RLT buffer plus β-mercaptoethanol and frozen on dry ice. Samples isolated from pools of 15 to 22 mammary primordia were used for each biological replicate.

### RNA purification

RNA samples were isolated using Qiagen RNeasy Micro Plus Kit (Qiagen, Hilden, Germany). Quality control, labelling and microarray hybridisation were performed at the Paterson Institute for Cancer Research, Molecular Biology core facility (Manchester, UK).

### Target amplification

Three biological replicates for each of the populations (micro-dissected mammary primordia, purified mammary primordial epithelium, purified mammary primordial mesenchyme) were amplified. Approximately 100 to 150 ng RNA was amplified with NuGEN amplification (NuGEN Technologies Inc., San Carlos, California, USA) and hybridised to MOE 430 2.0 Affymetrix arrays (Affymetrix Ltd, High Wycombe, UK).

### Primordial marker expression analyses

Immunohistochemistry (IHC) and whole-mount immunofluorescence (WM-IF) of E12.5 mammary primordia were performed as previously described [7,8]. Antibodies used in WM-IF or IHC are listed in Additional file 1. Whole mount *in situ *hybridisation of E12.5 mouse embryos and probes (sense and anti-sense) corresponding to nucleotides of 707-1050 NM_009242 for *Sparc*, 416-1139 of NM_023655 for *Trim29 *and 506-1206 of NM_032000 for *Trps1 *were generated as previously described [9].

### Quantitative real-time RT-PCR

To compare gene expression between the epithelium and mesenchyme at E12.5, total RNA was extracted from the purified mammary primordial compartments and three replicas were run with Taqman Array Assays-on-Demand probes on freshly isolated RNA (Applied Biosystems, Warrington, UK). Results were analysed with the Δ-ΔCt method normalised to *Actb*.

### Data analysis

Expression data were normalised and summarised by robust multi-array analysis (RMA) using the affy package in R. Probe sets were used for a multiclass Significance Analysis of Microarray (SAM) to determine if their mean expression was different across the three primordial populations. Two-class unpaired SAM was applied to determine genes enriched in one population when compared to another using a local false discovery rate (FDR) of 5%. Probe sets were used for a multiclass Significance Analysis of Microarray to determine if their mean expression was different across the three mammary epithelial cell (MEC) subpopulations described in [5] and the three primordial populations. A total of 4,000 probes identified in the multiclass SAM comparing the embryonic mammary populations with a FDR < 5% were clustered in R. Probes were centred by the median and clustered on the basis of their correlation distance using a Ward algorithm. In the embryonic-postnatal mammary analysis, the top 2,498 probes with a median absolute deviation (MAD) greater than two were clustered in R. Probes were centred by the median. Results were visualised in Java Treeview. Probes were considered population-enriched when they had a mean relative abundance of 1.5 or more. The microarray data have been submitted to the ArrayExpress repository with accession number [E-TABM-1099].

Network interaction maps were provided by uploading gene lists into a web-based in-house bioinformatics package and database, ROCK, developed based on the pSTIING server [10]. The core interaction maps were constructed using differentially expressed genes using only direct interactions characteristic of each population and produced one large interaction module for each tissue. Genes that did not directly connect are excluded. Interaction maps are displayed using ROCKscape with nodes representing proteins and edges representing interaction or transcriptional regulatory relationships between components. Sub-modules are identified based on observation of multiple interconnecting nodes within node groups that appear on the interaction map as separable node clusters. The interaction map generated by connecting the epithelial and mesenchymal populations was manually curated to ensure that interpolated connecting genes were appropriate (so that, for instance, transcription factors did not appear as nodes connecting genes across the epithelial and mesenchymal populations). Functional annotation for genes was determined using the Gene Ontology tool at the Mouse Genome Informatics website [11] and with the DAVID Bioinformatics Resource [12]. To determine the degree of similarity of gene expression profiles of mammary primordial cell populations and postnatal mammary and breast cells, genes considered characteristic of mouse postnatal mammary cells from [5] or the conserved mouse-human progenitor cells from [13] were compared to those of the embryonic mammary cells. The similarities are based on absolute present calls.

## Results

### Isolation of mammary primordial cell populations

**Figure 1:**
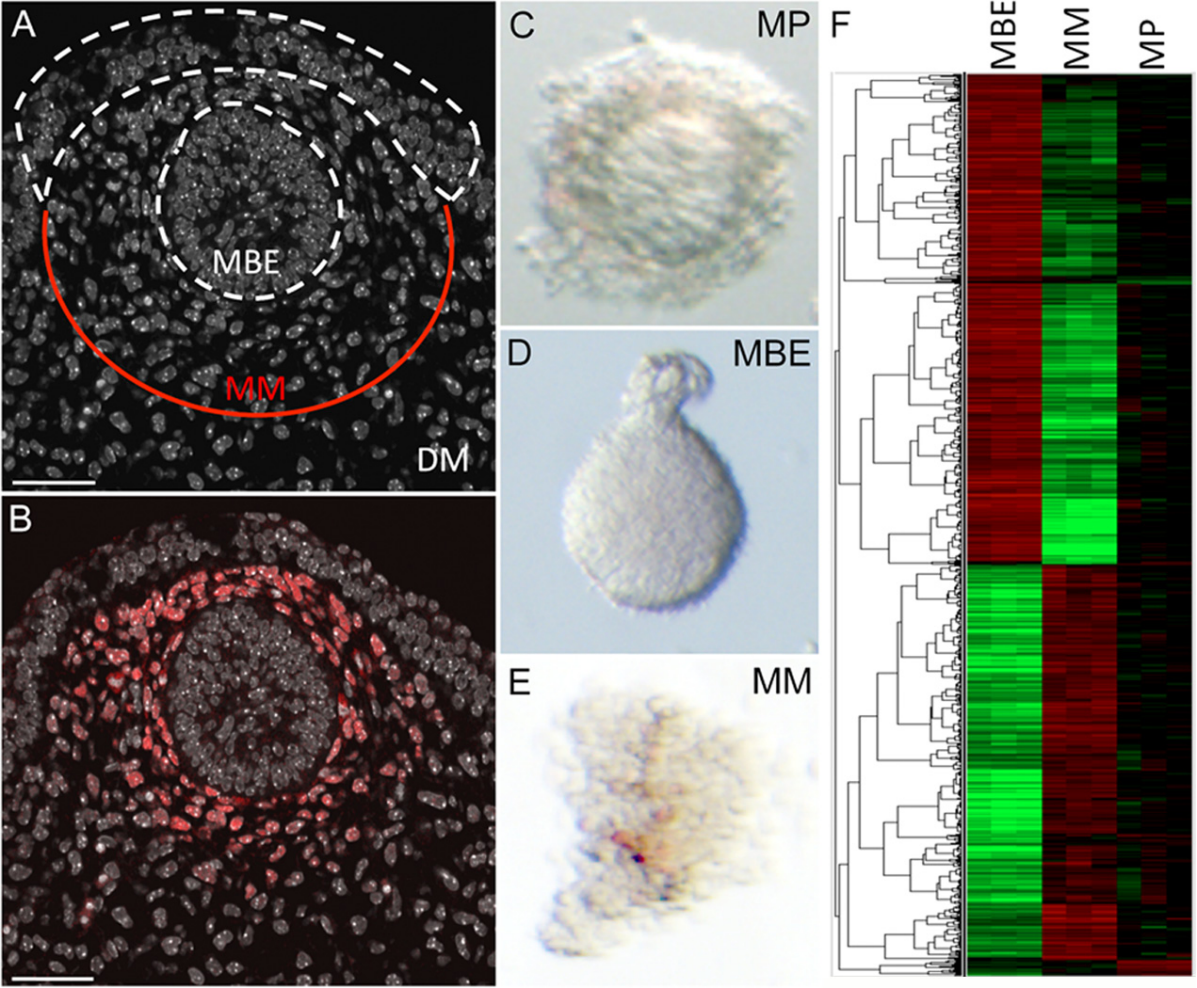
**Cell populations profiled to define the mammary primordial transcriptome**. **(A) **E12.5 mammary primordium stained with DAPI; the amount of mammary mesenchyme (MM) and dermal mesenchyme (DM) left associated with the mammary bud epithelium (MBE) upon microdissection are indicated. **(B) **Immunofluorescence with ERα shows stains throughout the mesenchymal tissue that was isolated. Scale bar is 50 μm. **(C) **Microdissected mammary primordium (MP). **(D) **Mammary bud epithelium after tissue separation. **(E) **Mammary primordial mesenchyme after tissue separation. **(F) **Heatmap and supervised clustering of differential gene expression across mammary primordial tissues with probe sets with the most tissue-specific expression. Each horizontal line represents a probe set with red indicating high and green indicating low expression.

Precise synchronised signals are thought to mediate the initial stages of mammary primordial development, since early stages of mammary morphogenesis progress in a highly stereotypical manner [14]. A limited amount of cell proliferation is observed within the mammary primordial epithelium during the early stages (E11.0 to E13.5) of mammary organogenesis. Localised cell movements are, therefore, thought to increase epithelial cell number during these stages of mammary development. To identify candidate signalling pathways and networks that are active during mammary organogenesis at a stage in which mammary inductive signalling and stem cell delimitation are occurring, we have generated data to define the mammary primordial transcriptome from stage E12.5 mouse embryos. This entailed micro-dissection of the fourth pair of mammary primordia from two or three litters of FVB/N embryos for each replicate (Figure 1A-C). In a second set of experiments, we separated the micro-dissected mammary primordia into their epithelial and mesenchymal cell populations (Figure 1D, E). In all experiments, the dissected mammary primordia included several layers of surrounding cells to gather the ERα-positive mesenchymal cells, which are arranged in layers of concentric circles around the mammary bud epithelium (Figure 1A, B).

### Gene expression profiling of mammary primordia and their epithelial and mammary mesenchymal cell populations

**Figure 2:**
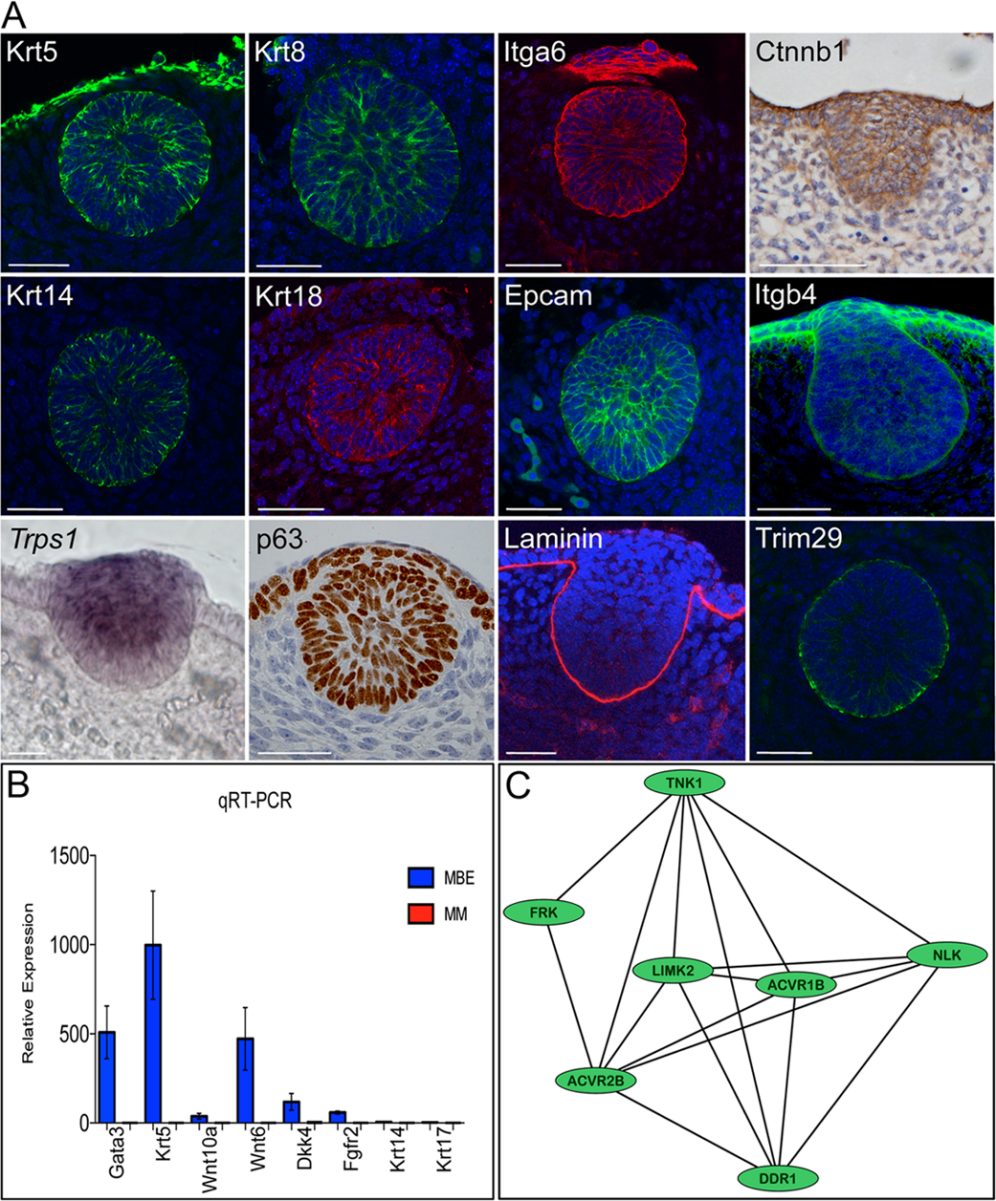
**Validation and network analysis of mammary primordial epithelium microarray data**. **(A) **Krt5, Krt14, Krt8, Krt18, Itga6/CD49f, and Epcam are expressed by many mammary primordial epithelial cells at high levels while some cells do not stain or express lower levels of these markers. All mammary primordial epithelial cells express p63. Laminin stains along the epidermal-mesenchymal boundary. Ctnnb1/β-catenin and Itgb4 are expressed at varying levels by cells within the primordia. Trim29 is expressed predominantly by basal cells. Scale bar is 50 μm. **(B) **qRT-PCR analysis of RNA from enzymatically separated E12.5 mammary bud epithelium (MBE) and mammary mesenchyme (MM). Gene RNA levels are expressed relative to *Actb *levels. Error bars ± s.e.m. Validation of eight mammary bud epithelium (MBE) specific genes showing enriched expression when compared to the mammary mesenchyme (MM). **(C) **A kinase-activin receptor sub-module is detected by network analysis. This sub-module forms part of the core network module generated using human orthologues of physically interacting genes characteristic of E12.5 mammary primordial epithelium. Black lines represent protein-protein interactions.

RNA was isolated from micro-dissected mammary primordia, and mammary primordial epithelium and mesenchyme from matched tissue pools. RNA was labelled by Nugen amplification and hybridised to MOE 430 2.0 Affymetrix arrays. Significance Analysis of Microarray (SAM) was applied to identify genes showing distinct expression patterns across the three different cell populations at an FDR < 0.05. Using these parameters, 4,000 probes were found to be differentially expressed among the enzymatically separated tissues and the micro-dissected primordia which were included to assess whether probes expressed by the separated tissues were present in the intact primordial samples (Figure 1F and Additional file 2). A total of 749 probe genes were upregulated 1.5-fold or greater at an FDR < 0.05 in the primordial epithelium compared to its associated mesenchyme when the mean relative abundance was 1.5 or greater (Additional file 2). Known key regulators of normal mammary primordial development, including *Edar, Fgfr2, Gata3, Msx1, Msx2, Nrg3, Pthlh*, and multiple Wnt signalling components were expressed specifically within the embryonic mammary epithelium when compared to the mesenchyme (Additional file 2) [15-23]. Amongst these, 25 genes were already known to be expressed in mid-gestation mammary primordia (Additional file 2). The technique used to separate the two tissues had relatively little effect on gene expression (Additional file 2). Using quantitative qRT-PCR, whole mount *in situ *hybridisation (WM-ISH), whole mount immunofluorescence (WM-IF), immunofluorescence (IF) and immunohistochemistry (IHC) we confirmed epithelial-enriched expression for 18 genes (Figure 2A, B). In addition, we detected expression of many other growth factors, receptors, and transcription factors not previously ascribed to the embryonic mammary epithelium (Additional file 3). RNA and protein expression studies have validated several population-enriched gene expression profiles detected by the array analysis and indicate the dataset represents a reliable global genetic profile of the primordial cell populations.

### Transcriptional network of mammary primordial development

A core network module (Additional files 3 and 4) was generated using ROCKscape, a modification of Cytoscape [24,25] by using the human orthologues of genes expressed by the E12.5 mammary primordial epithelium, as described in Additional file 2; only genes encoding proteins that have previously been shown to directly interact with each other were used to make this network. HEAT SHOCK PROTEIN 1 (*HSPB1*) is the mostly highly connected node and links to a keratin sub-module that is poised to maintain the structural integrity of the primordial epithelium. Other highly connected nodes include β-CATENIN (*CTNNB1*), an adherents junctions component and major effectors of Wnt signalling, PLAKOGLOBIN (*JUP*), PI3-KINASE (*PIK3R1*) and the actin-binding mediators, DUCHENNE MUSCULAR DYSTROPHY (*DMD*) and ACTIN-BINDING LIM PROTEIN 1 (*ABLIM1*). Less connected nodes include a kinase activin receptor sub-module (Figure 2C), a collagen-PDGF sub-module, an integrin/cell adhesion sub-module and a receptor-signalling sub-module. These sub-modules consist of genetic elements, many with established roles in the regulation of epithelial signalling and morphogenesis, including genes involved in focal adhesion, cell contractility, cell-matrix adhesion, and extracellular matrix (ECM)-receptor interactions. The sub-module in Figure 2C contains one node, *DISCOIDIN RECEPTOR 1 *(*DDR1*) which is known to regulate both branching morphogenesis and terminal differentiation in the postnatal mammary gland and is poised to interact with two activin receptors, represented by nodes *ACVR1B *and *ACVR2B *[26]. *Inhibbin-B*, a ligand for both activin receptors, is required during puberty and pregnancy for normal mammary ductal elongation and alveolar development [27]. This sub-module is, therefore, likely to represent biologically relevant interactions, since branching morphogenesis is a modified version of budding morphogenesis (that is, when a cluster of progenitor cells located within distinct boundaries moves, rearranges and proliferates to form an organ) and also identifies other kinases as novel potential bud morphogenetic regulators. Some signals within the epithelial cells should represent responses that have been induced by the mesenchyme (that is, activated receptors and their downstream effectors) as well as signals that will be sent to the mesenchyme (secreted factors).

A limitation of networks based on transcriptomic studies is that mRNA and protein levels do not always correlate. To address this to a small extent and further refine our understanding of the signalling characteristics of the primordial cells, we focused on validating expression patterns of markers within our network interaction maps using immunohistochemistry or immunofluorescence, rather than only qRT-PCR and ISH. Immunohistochemical analysis can be used to determine whether key nodes (based on number of interactions with other nodes and/or previous association with lineage) are present at the protein level and, therefore, are potentially signalling. This approach also permits detection of the precise location and fraction of cells within the mammary primordium that express putative effectors. One notable epithelial core network member, Trim29, appears to be restricted to the basal epithelial layer of the mammary epithelium at stages prior to the polarised postnatal localisation of the classic basal Keratins, Krt5 and Krt14, which are expressed by many basal and suprabasal mammary epithelial cells at E12.5. Trim29, therefore, represents one of the first mammary primordial markers expressed in a basally-enriched pattern, aside from the basal lamina components (Figure 2A). Trim29 can interact with Krt17, a keratin associated with stratification of epithelial appendages [28], which is expressed by basal cells of the postnatal mammary epithelia [5]. The location of Trim29 within the sub-module highlights a likely role in regulating processes related to both cell structure and lineage. In the postnatal mammary epithelium, keratins become predominantly segregated by expression within MECs of either the myoepithelial (Krt5, Krt14, Krt17) or luminal lineages (Krt8, Krt18).

**Figure 3:**
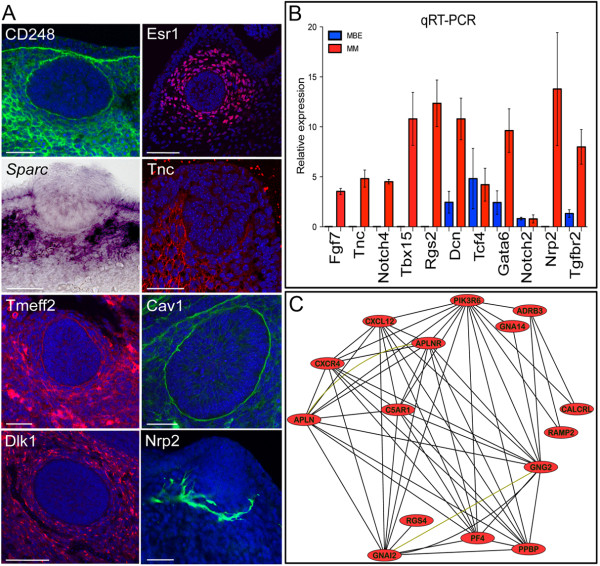
**Validation and network analysis of mammary mesenchyme microarray data**. **(A) **ERα and TnC stain the mammary mesenchyme. Most mammary mesenchymal cells express *Sparc*, except for those directly adjacent to the bud epithelium. Dlk1 and CD248/Endosialin stain all mesenchymal cells. Cav1 and Tmeff2 are expressed at higher levels by distinct cells. Nrp2 appears to be expressed by neural cells. Scale bar is 50 μm. **(B) **qRT-PCR analysis of RNA from enzymatically separated mammary bud epithelium (MBE) and mammary mesenchyme (MM). Gene RNA levels are expressed relative to *Actb *levels. Error bars ± s.e.m. Validation of nine MM specific genes enriched expression when compared to the MBE. *Tcf4 *did not show population specific expression by this analysis. **(C) **A sub-module comprised of several highly interconnected nodes representing G protein coupled receptor signalling and cytokines forms part of the core network module of mammary primordial mesenchyme. This core network was generated using human orthologs of directly interacting genes characteristic of the mammary mesenchyme. Black lines represent protein-protein interactions. Nodes that have been shown to interact in complexes are connected together by yellow-green lines.

Population-enriched expression was observed for 642 probes at FDR < 0.05 in the mammary mesenchyme associated with the mammary primordium when compared to the epithelium when the mean relative abundance was 1.5 or greater (Additional file 2). We detected the previously reported mesenchymal-enriched expression of *Fgf7, Tbx15*, and *Tnc *[17,29-31]. We confirmed mesenchymal-enriched expression for 13 genes using qRT-PCR, WMISH, and WM-IF (Figure 3A, B). Major transcriptomic pathway components expressed specifically within the mammary mesenchyme are summarised in Additional file 5, including many growth factors, receptors, and transcription factors. A core network module (Additional file 5 and 6) was generated using ROCKscape based on the human orthologues of directly interacting genes characteristic of the mesenchyme associated with the E12.5 mammary primordial epithelium listed in Additional file 2. A highly interconnected sub-module comprised of G protein coupled receptor signalling and cytokine signalling is evident and contains key mediators of metastasis and invasive growth, such as CXCR4 and its ligand CXCL12/SDF [32] (Figure 3C). This sub-module connects to another highly interconnected sub-module comprised of cytoskeleton and cell movement components via the node, GELSOLIN, *(GSN)*. The actins binding protein, (*Gsn*), is required in postnatal stroma for proper mammary ductal elongation into the fat pad during puberty and this phase of invasive growth is very delayed in its absence [33]. In addition to suggesting a possible role for *Gsn *in the regulation of embryonic mammary epithelial morphogenesis, the network analysis identifies connections to plausible interaction partners since both CXCR4 and CXCL12/SDF have well-established roles in invasive cell behaviour. Many mesenchymal nodes represent genes involved in angiogenesis, chemotaxis, and neural pathfinding (Additional file 6). One node, NEUROPILIN2 (*NRP2)*, is highly expressed in the mammary mesenchyme array data and was validated by qRT-PCR (Figure 3B); Nrp2 appears to stain nerves (Figure 3A) whilst other markers appear to stain forming blood vessels in the mammary mesenchymal region that was dissected out and profiled (Figure 1A). This indicates that despite its indistinct and homogenous cellular appearance, mammary mesenchymal components (neural, vasculature, fat cells) indicated by signature, pathway, and network analyses are likely to reflect ongoing developmental processes associated with specific cell types. Some mesenchymal markers are expressed in sub-domains that could represent or promote the formation of distinct sub-compartments within the mammary mesenchyme.

### Potential mammary-inducing factors, lineage promoters and key signalling processes identified

By E13.0, embryonic mammary mesenchyme from mice can induce surface epithelium to adopt a mammary cell fate [34], so E12.5 mammary mesenchyme should be enriched for mammary inducing factors. Postnatal stromal cells are not thought to harbour mammary inductive potential. Factors with the potential to transmit mammary-inductive signals were identified by analysing gene expression profiles enriched within the mammary mesenchyme compared to stromal signatures described in [13]. Several secreted factors including *Dlk1 *and *Gas1*, fulfil this criterion and as such are potential mammary-inducing factors (Additional file 7).

To identify potential promoters of mammary differentiation from the inception of the mammary lineage onwards, we compared genes expressed by the mammary mesenchyme with genes expressed by the mammary stroma profiled by [13] (Additional file 8). Genes expressed by both tissues could promote mammary differentiation throughout mammary development and act as lineage reinforcers to maintain mammary cell identity. Several secreted factors are classified as potential mammary lineage reinforces including, *Ccl11, Dab2, Dcn, Igf1, Fgf7, Figf, Gpc3, Sparcl1, Slit2, Thbs2, Tmeff2*, and *Wisp1 *(Additional file 8). These molecules could signal directly to the primitive and mature MECs to promote mammary cell fate. In addition, several transcription factors also fall into this category, including *Ebf1, Ebf3, Gata6, Klf4, Maf, Nr2f1, Osr2, Prrx1, Prrx2, Tcf4, Tbx15*, and *Tbx18*, which may contribute to promotion of the mammary cell fate via transcriptional regulation of other particular key factors that can transmit signals to the adjacent MECs. Comparison of genes expressed by the mammary mesenchymal cells with genes expressed by human breast stromal cells identifies some common genes (Additional file 8). This may indicate a conserved function of some stromal signals across species with respect to mammary lineage promotion and maintenance.

**Figure 4:**
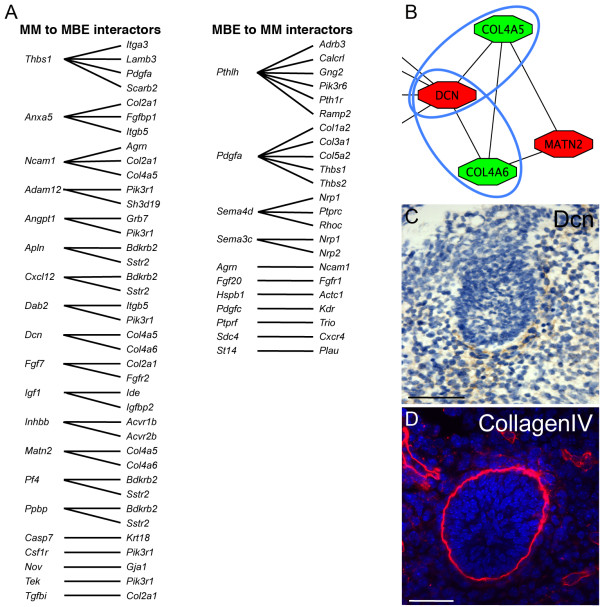
**Prospective mediators of epithelial-mesenchymal interactions identified by network analysis**. **(A) **List of likely effectors of inductive signalling across the mesenchymal-epithelial boundary. In addition to ligand-receptor pairs, inter-connections between known signalling cascade components that could transmit signals across the mammary mesenchyme (MM) and the mammary bud epithelium (MBE) and *vice versa *are shown. **(B) **Mesenchymal Decorin (Dcn) is poised to interact with both epithelial CollagenIVa5 and CollagenIVa6 (Col4a5 and Col4a6). Red represents mesenchymal nodes. Green represents epithelial nodes. **(C, D) **Decorin and CollagenIV expression patterns suggest that these interconnected nodes represent feasible biological interactions that could modulate signals both within the extracellular matrix and basement membrane and across the mesenchymal-epithelial boundary. Scale bar is 50 μm.

The mammary primordial epithelial and mesenchymal core network modules were interconnected to identify prospective mediators of mesenchymal-epithelial interactions (Additional file 9). Some of the putative mammary inductive signals expressed by cells in the mammary mesenchyme are found in the core interconnected network module and plausible signalling partners or signalling components have been identified in the mammary bud epithelium (Figure 4A, B). For example, Decorin (Dcn) is expressed by mesenchymal cells immediately adjacent to the mammary bud epithelium (Figure 4C). Decorin, a proteoglycan, is secreted and thus poised to interact with epithelial cells expressing Col4a5 and Col4a6 (CollagenIV) at the epidermal-mesenchymal boundary (Figure 4D). Studies of *Dcn*-null mice have identified a role for *Dcn *in the regulation of collagen fibril assembly which is thought to lead to the formation of a barrier to cell penetration and macromolecule infiltration [35]. Decorin has also been implicated in the modulation of both postnatal mammary stromal cell phenotypes and MEC behaviour in tumours [36], suggesting that Decorin may play a regulatory role in mediating mammary tissue interactions from early developmental stages onward. Established regulators of mesenchymal to epithelial signalling such as *Inhbb *(activin/inhibin beta b) are represented in this network (Additional files 9 and 10). Several secreted factors are found that could interconnect nodes to mediate signal transduction across the two tissue compartments via paracrine signalling (Figure 4A). Many interactions that are poised to connect the epithelial and mesenchymal cells (by virtue of their compartment-enriched expression and known ability to interact) could modulate extracellular matrix organisation and cell adhesion and would, therefore, profoundly alter the microenvironment of progenitor cells in the basal layer of the epithelium.

### Comparison of gene expression profiles of mammary primordial cell populations reveals similarities and differences with their direct descendants

**Figure 5:**
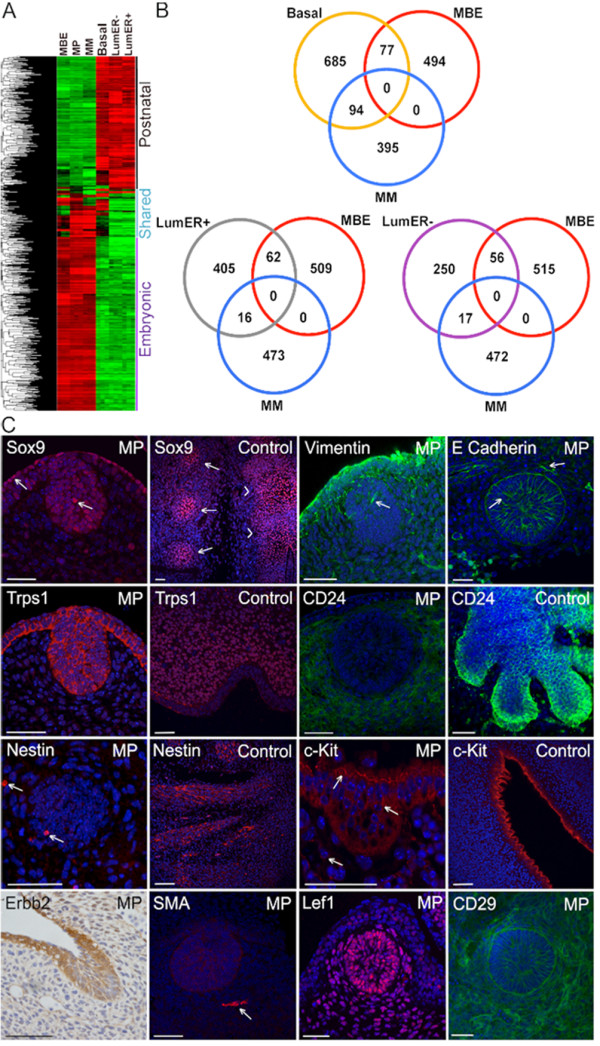
**Transcriptome analysis reveals similarities and differences between embryonic and postnatal mammary cell populations**. **(A) **Heatmap and unsupervised cluster analysis of differential gene expression across postnatal mammary epithelial cells (MECs) and mammary primordial populations. Each horizontal line represents a probe set with red indicating high expression and green indicating low expression in the various tissue compartments. **(B) **Venn diagrams showing the number of shared and unique genes expressed within mammary primordial compartments compared to the three postnatal mouse MEC subpopulations. **(C) **Arrows indicate cells expressing nuclear Sox9 within the surface and primordial epithelium. Control tissue shows strong nuclear staining in the developing ribs (arrows) and spinal cord (arrowheads). Strong Vimentin expression is observed in the periderm, surface epithelium and mammary mesenchymal cells. Arrow indicates a mammary primordial epithelial cell expressing Vimentin. Most suprabasal cells of the mammary primordial epithelium express E-cadherin; less intense expression is observed in the periderm and surface epithelium above the mammary bud and some suprabasal cells (arrows). Trps1 stain is non-nuclear within the mammary bud epithelium. Vibrissae mesenchyme shows nuclear stain while some non-nuclear staining is observed in the epithelium at E12.5 (Control). Diffuse CD24 is observed in E12.5 mammary mesenchyme. At E16.5, the mammary bud epithelium and some cells within the mammary mesenchyme express CD24 (Control). A few Nestin-expressing cells are observed within both mammary epithelial and mesenchymal primordial cells. Strong Nestin staining is observed in embryonic nerves at E12.5. Some c-Kit positive cells are observed within the mammary bud epithelium and mesenchyme. Positive control is roof of brain ventricle at E12.5. Erbb2 expression is observed in mammary bud epithelial and some mesenchymal cells at E12.5. SMA is observed in a blood vessel (arrow) adjacent to the mammary bud epithelium, which does not express SMA. Both the mammary bud epithelial and mammary mesenchymal cells express Lef1 and CD29. Scale bar is 50 μm. MBE denotes mammary bud epithelium. MM denotes mammary mesenchyme. MP denotes mammary primordium. LumER- denotes luminal estrogen receptor negative. LumER+ denotes luminal estrogen receptor positive.

The mammary primordial populations profiled were isolated from the fourth primordial (of the five pairs) from the FVB strain so a direct comparison to the postnatal mammary epithelial cell sub-populations (basal, luminal ER-, luminal ER+) was possible which were also isolated from the FVB strain and the fourth (inguinal) mammary gland (Figure 5A and Additional file 11) [5]. As such, the postnatal cells should represent the descendants of the primordial cells. Three major classes of genes can be identified: those expressed within the postnatal cell populations and not expressed by the primordial cells (postnatal mammary), those expressed within the primordial cells and not expressed by the postnatal cells (embryonic mammary), and those that are expressed by both the prenatal and postnatal mammary cell populations (shared embryonic/postnatal mammary) (Figure 5A). Gene expression patterns shared between primordial and postnatal mammary sub-populations may represent effectors capable of impacting mammary cell behaviour regardless of their level of differentiation (Figure 5B and Additional file 12). The mammary primordial epithelial cells are an immature mammary epithelial cell population and lack markers of differentiated cell types, such as ERα, which is expressed by cells in the luminal ER+ population or SMA which is expressed by myoepithelial cells which comprise the bulk of the basal population (Figures 1B and 5C) [37].

We also compared our embryonic mammary gene signatures to the terminal end bud (TEB) and mature ductal signatures derived from the dataset described by Kouros-Mehr and Werb [38]. This dataset represents profiles of micro-dissected tissues containing both epithelial cells and immediately adjacent stroma at puberty, a key developmental stage during which the TEB exhibits invasive directional growth through the fat pad, resulting in ductal elongation and branching. We found a small number of genes expressed by embryonic mammary cells and ductal or TEB cells, including *Anxa8, Dlx3, Kitl, Klf4, Pthlh, Sema3c. Sox11, Sox18, Thbs1, Trps1, Wnt6*, and *Wnt7b*. Genes expressed at both of these critical developmental stages may represent master mammary morphogenetic or lineage regulators (Additional files 12 and 13). Of these, some are also expressed by human stromal cells including *Col3a1, Fbn1, Igf1, Kera, On, Ptgis, Thbs2, Timp3, Tmeff2*, and *Vcam1*.

By E13.0, the mammary primordial epithelium is determined to mammary cell fate, so at this slightly earlier developmental stage, E12.5, profiled here, the primitive mammary epithelium is either stably or nearly determined to the mammary lineage and, therefore, genes regulating mammary progenitor cell function should be expressed. Since other organs express the same transcription factors in stem/progenitor cells at both embryonic and postnatal stages, we looked for shared transcriptional programs across the embryonic and postnatal mammary cells to ascertain potential tissue-specific transcription factors and other signalling regulators, which we predict should be involved in mammary cell fate and progenitor function (Additional file 13). Examples of transcription factors that are expressed in both primordial and postnatal MEC sub-populations and have critical roles in regulating progenitor biology include *Elf5 *and *Sox6 *[5,39]. Several mammary stem cell-enriched and progenitor-enriched markers are expressed by the mammary primordial cells (Figures 2A, 5C and Additional file 13).

Comparison of the primordial populations to the postnatal mammary cell populations reveals subsets of genes that are expressed within both the mammary primordial epithelium and associated mesenchyme (Figure 5A and Additional file 13). Some of these include classic EMT genes, including *Twist1 *and *Twist2*, which are associated with loss of epithelial characteristics when expressed by adherent epithelial cells and acquisition of mesenchymal features (Additional file 13). Confocal analysis of the mammary primordium shows a very heterogeneous population of cell types, some with a nucleus and shape resembling fibroblasts (Figure 1A). We have found that a small population of the primitive mammary epithelial cells express an epithelial-mesenchymal transition (EMT)-associated marker Sox9 and Vimentin, a mesenchymal marker expressed by some *bona fide *epithelial cells, as well as those undergoing EMT (Figure 5C). Myoepithelial cells also express EMT-associated genes so their expression by the various mammary cell populations is likely to reflect their shared developmental origin from the basal epithelium, including an underlying transcriptional program that acts to repress epithelial differentiation and promote cell motility. Primordial epithelial cells with EMT features may have great pertinence to the initial generation of mammary stem cells since recent data suggest that induction of EMT stimulates cultured breast cells to adopt characteristics of stem cells [40].

### Comparison of gene expression profiles of mammary primordial cell populations reveals transcriptomic similarities to human breast progenitor profiles

We also compared genes expressed in both the mammary primordium and the subpopulations of MECs that are conserved across mouse and human, which have recently been described [13]. We observed some overlapping gene expression with the primordial cell populations and the "mammary stem cell (MaSC)" populations, luminal progenitor and mature luminal populations (Additional file 13). Genes with established or anticipated roles in mammary progenitor biology, including *Itga6 *and *Msx1*, are expressed by the mammary primordial cells, and by the conserved mouse-human mammary progenitor populations (Additional file 14). Many genes expressed by both the primordial and conserved mouse/human mammary stem cell-enriched populations are also found within the core network modules which suggests that the primordial core networks reflect elements regulating fundamental biological processes, with many poised to impact progenitor cell behaviour.

### GSEA detects similarities to other progenitor populations

Gene expression profiles of the embryonic and mammary postnatal cells are largely unique with a limited amount of overlap (Figure 5A, B). Primordial population signatures were used in gene set enrichment analysis [41]. When compared to the luminal postnatal MEC subpopulations, the mammary primordial epithelium displays enrichment for Wnts, Hox genes, phosphatidylinositol, insulin signals, and gene signatures found up-regulated in many hematopoietic progenitor classes. When compared to the basal MECs, the mammary primordial epithelium is enriched for Hox genes, phosphatidylinositol, and insulin signals, but is not enriched for progenitor cell signatures or Wnt signals (Additional file 15). This indicates that the embryonic mammary cells share some genetic features with other progenitor cells, particularly hematopoietic types.

## Discussion

The results presented here give a global picture of the signalling events that may occur as the mammary lineage is first established during mammary primordial organogenesis. Mammary primordial epithelial cells express a distinct entity of genes when compared to those present within the adjacent mammary mesenchymal cells (and *vice versa*). We have used these gene signatures to define the transcriptomic specificity and signalling cascades that are likely to be mediating reciprocal epithelial-mesenchymal interactions that regulate early mammary tissue organisation, architecture, differentiation and cell fate decisions. Inducing factors such as Nrg3, Eda, Fgf, and Wnt signals have been shown to promote either mammary cell fate and/or epidermal appendage development and key components of these pathways are detected in our analysis [17,19,20,42]. The network maps show how many of the genes expressed by the mammary primordium might converge to elicit complex processes required for morphogenesis such as changing cell adhesion to allow local cell movements required during budding morphogenesis.

Since mammary mesenchyme is an instructive tissue, a strong component of its cell function should be to promote mammary lineage establishment. The mammary primordial epithelial cells profiled here should be receiving signals from the mesenchyme representing key aspects of mammary cell fate acquisition. Comparing gene expression enriched within mammary mesenchyme to that enriched within postnatal mammary stoma identified factors with potential to transmit mammary-inducing signals. Notable candidates with potential mammary-inductive signalling roles include *Gas1*, which regulates the range and the level of Hedgehog signalling, since down-regulation of Hh signalling is absolutely required for mammary development to proceed [43-45]. *Dlk1*, an endogenous inhibitor of Notch, could act to repress Notch signals in the mammary primordial epithelium; Notch controls cell fate and tissue homeostasis in postnatal mammary epithelium and could be regulating stem cell function from the time that they arise [46]. Other genes of interest include *Arhgap28 *and *Centd3 *since another Rho-GTPase activating protein-encoding gene, *Arhgap5*, has been shown to regulate epithelial-mesenchymal interactions necessary to sustain mammary bud morphogenesis [47].

This approach to classifying molecules may be useful for identifying likely mediators of mammary inductive signalling for further study but all diffusible factors expressed by the mammary mesenchyme are potential mammary inducing factors. Signals from purified mammary mesenchyme (isolated using similar methods used in this study) can induce mammary glandular tissue when recombined with simple epithelium across species (mouse to rat) and (rabbit to chicken and duck) so the putative paracrine interactions identified here are likely to occur in other species, including human [34,48]. A recent study showed that embryonic prostate mesenchyme acts to induce prostate lineage markers in tissue recombinants using several types of non-prostate postnatal epithelium [49]. This notable study supports the contention that mesenchymal and stromal tissues act as lineage enforcers which send signals to epithelial cells to promote and maintain differentiation along a specific lineage. The interconnected epithelial-mesenchymal nodes in our network map predominantly involve signals that involve basal lamina and ECM components and many, such as the integrins, have established roles as cell differentiation and cell adhesion regulators which impact cell shape, changes in cell contact, and cell movements, consistent with the morphogenetic processes that these tissue interactions invoke.

Epithelial *Pthlh *to mesenchymal *Pthr *signalling is required for the formation of the mammary mesenchyme and the induction of *Tnc *expression [29]; *Pthlh *represents the most abundant gene expressed by the embryonic mammary epithelium in our dataset. A recent study profiling genes expressed by micro-dissected E15.5 stage *Pthlh-/- *and wild-type mammary primordia, to study ductal morphogenesis, detected many changes in genes expression by the *Pthlh *mutant primordia, consistent with *Pthlh *signalling being a key component of mammary primordial development [50]. However, this analysis did not permit the identification of which of the two tissues the candidates genes were expressed within since the primordia were not separated into their two tissue components. We detected expression of several of the candidate genes that appear to be regulated by *Pthlh/Pthr *signalling in our core interconnected mammary primordial network module (including *INHBB, KRT8, KRT18, KRT19, MATN2, SOSTDC1, TGFBI, THBS1, NRP1, WISP1 *and *TNC*) giving a more precise indication of how this signalling cascade could proceed and connect diverse pathways across the tissues to control reciprocal mesenchymal-epithelial *Pthr/Pthlh *signals. Our core network reflects signals from an earlier developmental stage (E12.5) at which it is conceivable that *Pthlh *and *Pthr *signals are already actively inducing formation of the mammary mesenchyme. The *Pthlh/Pthr *regulated genes identified by [50] found in our E12.5 networks are, therefore, likely to reflect some key elements that confer or maintain instructive properties to the mammary mesenchyme. This indicates our datasets and networks represent profound ongoing inductive events and should be useful for identification of novel putative inductive signalling effectors, which can be tested for roles as embryonic mammary development regulators using either transgenic or knockout mouse models, which should soon be available for most genes via ongoing large-scale mouse genome initiatives. As more genes and pathways are determined to regulate embryonic mammary primordial development, these networks can be extended and expanded which will permit assessment of potential genetic hierarchy and pathway convergence and aid experimental design to clarify these issues.

Although the mammary primordium should in theory be predicted to be enriched for stem cells, previous functional analysis suggests that only a very small fraction of the primordial epithelial cells can fulfil the standard definition currently used to assay for mammary stem cell function, that is, the repopulating capacity in the cleared postnatal fat pad [4]. It is possible that this assay is not particularly appropriate for establishing stem cell activity of primordial cells since it selects for more mature stem cells and may exclude less-mature embryonic progenitor cells that are incapable of interacting with the postnatal microenvironment yet fulfil stem cell criterion within their native microenvironment *in vivo *(that is, they undergo self-renewal and are capable of giving rise to other cells types). Results from gene set enrichment analysis indicate that the primordial cell populations share some transcriptomic features with other progenitor populations from both embryonic and postnatal tissues, particularly hematopoietic progenitors. Recently, s-SHIP promoter expression has been shown to mark activated stem cells in postnatal mammary tissue [51]. It is, therefore, notable that mammary primordial epithelial cells express GFP driven by the s-SHIP promoter [52], which indicates that the mammary primordial epithelial cells may represent a population of activated primitive mammary progenitor cells. Although by E13.0, the mammary epithelium is determined as mammary, it is highly likely that a large degree of plasticity exists with respect to how committed, (if at all) any of the individual primordial cells are to any particular progenitor type (stem, bipotent, myoepithelial precursor, luminal precursor) within the mammary hierarchy at this stage of development and remains to be explored.

Our results show that substantial transcriptomic differences exist between embryonic mammary cells and postnatal mammary cells, which are most likely due to a unique microenvironment and their immature state. Despite this, the primitive embryonic epithelial and mesenchymal cell populations express a number of genes that have been shown or suggested to regulate postnatal progenitor mammary cell functions. The conserved expression patterns observed across the primordial and postnatal MEC sub-populations suggest that some molecular mechanisms that govern mammary stem cell homeostasis are active as the lineage is first established and continue to regulate this function throughout postnatal development. The early embryonic expression of several known mammary lineage regulators within the primitive mammary epithelium lends support to this idea. In particular, lack of *Gata3*, one of the most highly expressed genes expressed by the embryonic mammary epithelium in our analysis, leads to defective mammary primordial development at stages prior to E12.5 [21].

## Conclusions

The pathways implicated in cell fate decisions are clearly relevant to transformation as most forms of breast cancer involve deregulation of signalling pathways (Egg, Fgf, Notch, Wnt) that also determine cell identity when cells are initially specified from undifferentiated precursor cells [53]. Embryonic mammary developmental studies will be key to determining how the mammary primordial cells may differentiate and perpetuate amidst a variety of other cell types and signals to first establish an environment or niche for the penultimate mammary progenitor cells. Functional studies of the mammary mesenchyme have demonstrated its profound role in directing and maintaining normal mammary tissue differentiation and architecture; mammary mesenchyme can restore some features of differentiated tissue to mouse mammary tumours when grown together in culture [54]. The identification of molecular effectors that may contribute to the creation of the primitive mammary epithelial and stromal populations provides a basis for selection of novel candidate regulators of mammary lineage establishment, maintenance, and differentiation for further study in both mouse models and cell-based systems. The dataset and analysis presented here represent a framework for studying how cells communicate and act together to produce the organ that defines and sustains all mammals.

## Abbreviations

Dcn: Decorin; ECM: extracellular matrix; EMT: epithelial-mesenchymal transition; ER: estrogen receptor; *HSPB1*: HEAT SHOCK PROTEIN 1; IHC: immunohistochemistry; Krt: keratin; MAD: median absolute deviation; MaSC: mammary stem cell; MBE: mammary bud epithelium; MEC: mammary epithelial cell; MM: mammary mesenchyme; MP: mammary primordium; qRT-PCR: quantitative real-time RT-PCR; RMA: robust multi-array analysis; SAM: Significance Analysis of Microarray; SMA: smooth muscle actin; TEB: terminal end bud; WM-IF: whole-mount immunofluorescence; WM-ISH: whole mount *in situ *hybridisation.

## Competing interests

The authors declare that they have no competing interests.

## Authors' contributions

OW isolated RNA, performed *in situ *hybridisations, IHC, IF and carried out qRT-PCR analysis. AM carried out SAM analysis on the array data and processed the datasets. NK isolated, stained and analysed mammary primordial samples and performed gene set enrichment analyses. KD and CR contributed samples, antibody staining, and manuscript writing and review. HK provided samples and assisted with qRT-PCR analysis. JSR-F and MJS contributed to the design of the study, manuscript writing and review. CM and MZ carried out network analysis on array data. BAH conceived the study, collected primordial samples, analyzed the microarray data and wrote the manuscript. All authors have read and approved the manuscript for publication.

## Supplementary Material

Additional file 1 **Antibodies used in expression analysis experiments**. Table gives details of antibodies used in this study. Click here for file

Additional file 2 **The mammary primordial transcriptome**. Table A displays relative expression levels of 4,000 Affymetrix probes across three embryonic mouse mammary primordial populations. The spreadsheet gives the relative expression levels for all differentially expressed probes across all three populations. Expression levels are indicated by a relative abundance score for each population. A high positive value indicates expression at a high level, a low negative score indicates very low expression levels. The Affymetrix probe ID, Gene Symbol and q-value (indicating the % false discovery rate) are also indicated. Table B contains genes characteristic of mammary primordial epithelial cells. The table shows all 749 probes in the mammary primordial epithelial population with an abundance score of 1.5 or more when the differentially expressed gene set was sorted by descending abundance scores in the epithelial population. Such genes were considered population-enriched. The number of probes is indicated. Table C contains probes with known expression patterns that were detected as epithelial-specific or epithelial-enriched expression in the arrays. Table D contains probes induced or repressed by tissue separation. Table E contains genes characteristic of mammary primordial mesenchymal cells. The table shows all 642 probes in the mammary primordial mesenchymal population with an abundance score of 1.5 or more when the differentially expressed probe set was sorted by descending abundance scores in the primordial epithelial population. Such genes were considered population-enriched. Click here for file

Additional file 3 **Transcriptomic characteristics of the mammary primordial epithelium**. **(A) **A figure summarising select genetic components of the mammary primordial epithelium detected by array analysis. **(B) **A figure depicting network analysis of the mammary primordial epithelium. Click here for file

Additional file 4 **Mammary epithelial primordial core network members**. Table contains mammary epithelial core network members. Click here for file

Additional file 5 **Transcriptomic characteristics of the mammary mesenchyme**. **(A) **A figure summarising select genetic components of the mammary mesenchyme detected by array analysis. **(B) **A figure depicting network analysis of the mammary mesenchyme. Click here for file

Additional file 6 **Mammary mesenchymal primordial core network members**. Table contains mammary mesenchymal core network members. Click here for file

Additional file 7 **List of candidate mammary inducing factors with Gene Ontology**. This table contains expression patterns of genes enriched in primordial-associated mesenchyme when compared to postnatal mammary stroma in a previously published dataset [13]. Gene expression patterns were overlaid in order to discern enriched secreted factors present only in the mammary mesenchyme that could confer mammary cell phenotype to uninduced surface epithelium. Click here for file

Additional file 8 **Lists of candidate mammary lineage promoting/enforcing factors**. Table **A **contains genes expressed by both the mammary mesenchyme and the mature mammary stroma in a previously published dataset [13]. These genes are candidates for promoting mammary differentiation or enforcing mammary lineage commitment. Table **B **contains genes expressed by human mammary stromal cells in a previously published dataset [55] which were also found expressed by the mammary mesenchymal cell populations in the current study. Click here for file

Additional file 9 **Prospective mediators of epithelial-mesenchymal interactions identified by network analysis**. **(A) **A figure depicting the core interconnected network module. **(B) **A figure depicting how mesenchymal INHBB is poised to bind to epithelial ACVR1B and ACVR2B. Click here for file

Additional file 10 **Core interconnected network members with Gene Ontology**. Table contains core interconnected network members with Gene Ontology. Click here for file

Additional file 11 **Multiclass SAM analysis of embryonic and postnatal mammary populations**. This table displays relative expression levels of 16,382 Affymetrix probes across three embryonic mouse mammary primordial populations and three postnatal mammary epithelial populations. The spreadsheet gives the relative expression levels for all differentially expressed probes across all six populations. Expression levels are indicated by a relative abundance score for each population. A high positive value indicates expression at a high level, a low negative score indicates very low expression levels. The Affymetrix probe ID, Gene Symbol and q-value (indicating the % false discovery rate) are also indicated. Click here for file

Additional file 12 **Genes expressed by embryonic mammary cells and postnatal mammary cells**. This table contains genes common to primordial cells and basal cell dataset and luminal cell datasets from [5]. The table lists those basal genes, LumER- genes, and LumER+ genes found in a previously published dataset [5] which were also found in the primordial epithelial or mesenchymal populations in the current study. A mouse stromal signature from [13] was used to determine which genes were also found in the primordial epithelial or mesenchymal populations. Genes expressed by embryonic mammary primordial cells and the cells contained within the terminal end bud and ductal microenvironments from [38] are also listed. Click here for file

Additional file 13 **Comparison of gene expression profiles of embryonic mammary cells with postnatal mammary cells**. **(A) **Venn diagrams showing comparison of genes expressed by embryonic mammary primordial cells to those expressed by the cells contained within the terminal end bud and ductal microenvironments from [38]. **(B) **A heatmap showing the expression of a variety of key developmental, lineage, and progenitor markers in embryonic and postnatal mammary cell populations. **(C) **Venn diagram showing comparison of genes expressed by embryonic mammary primordial cells to those expressed by the conserved mouse/human MEC subpopulations and human fibroblasts from [13,55]. Click here for file

Additional file 14 **Genes expressed by embryonic mammary cells and human breast cells**. This table contains genes common to primordial cells and conserved mouse/human mammary stem/myoepithelial cells, luminal progenitors or mature luminal cell dataset from [13]. The table lists those conserved mouse/human genes expressed by mammary stem cell (MaSC) or myoepithelial cells, luminal progenitors (lum prog), and mature luminal (lum mature) cells found in a previously published dataset [13] which were also found expressed by the primordial epithelial (MBE) or mesenchymal (MM) populations in the current study. The human stromal signature defined in [55] was used to determine which genes were also found in the primordial epithelial or mesenchymal populations. Click here for file

Additional file 15 **Gene set enrichment analysis of mammary primordial gene expression data**. This file contains the gene sets (with a nominal *P-*value of < 0.05 in order of significance) enriched in primordial cells and postnatal cells when the two data sets were compared. Gene sets with similarity to progenitor populations are in italics and underlined. Gene sets with a FDR q value of < 0.50 are in bold. Click here for file
