# Supplementary material for: Transcriptome analysis of embryonic mammary cells reveals insights into mammary lineage establishment
Source: Breast Cancer Res. 2011 Aug 11;13(4):R79. doi: 10.1186/bcr2928 (PMC3236343; doi:10.1186/bcr2928)
Supplement: Additional file 3 — Transcriptomic characteristics of the mammary primordial epithelium. (A) A figure summarising select genetic components of the mammary primordial epithelium detected by array analysis. (B) A figure depicting network analysis of the mammary primordial epithelium. [file bcr2928-S3.PDF]

A

Select mammary bud epithelial genes

Wnt pathway: *Wnt3, Wnt6*, Wnt7a, Dkk4*, Wnt10a*, Fzd6, Wnt10b, Wnt7b, Wnt4, Frz10, Fzd3, Ctnnb1**

Bmp pathway: *Bmp7*, ***Msx1***

Erbb pathway: ***Nrg3,*** *Erbb3, Errfi1*

Fgf pathway: ***Fgfr2****, Fgf20, Fgfbp1, Fgf17*

Tnf pathway: *Edar, Edaradd, Tnfaip2, Traf3, Tnfrsf19*

Transcription factors: ***Gata3*****, Elf5,* ***p63*****, Grnhl2, Pou3f1, p73, Sox6, Sp6, Dach2, Runx1, Sox9*, Six4, Fxyd3, Ap-2 alpha, beta, & gamma, Sox21, Klf5, Lmo1, Foxq1, Rhox4b, Dach1, Sox11, Sox13, Mycn, Trps1**

Homeobox-containing transcription factors: ***Msx1***, ***Msx2****, Hoxc5, Hoxc8, Irx4, Irx6, Irx5, Dlx3*

Cytokines: *Cxcl15, Cxcl14*

TGF pathway: *Acvr2b, Acvr1b*

Notch pathway: *Jag2, Nrarp, Notch1*

IGF pathway: *Igfbp2*

Axon guidance: *Sema3e, Sema3d, Sema3c, Unc5b, Sema4d, Epha1*

Keratins : epidermal *Krt8*, Krt18*,*

periderm *Krt19, Krt7;*

epidermal stratification *Krt17*, Krt1, Krt5*, Krt14**

Desmosomal components: *Dsp, Dsc2, Dsg2, Pkp1, Pkp3, Ppl*

Tight Junction components: *Ocln, Cldn7, Cldn23, Cldn8, Cldn3*

Cadherins: *Pcdh1, Celsr1, Pcdh8, Cdh1*, Cdh3, Celsr2, Cdh13, Pcdh7, Pcdhb16*

Collagens: *Col4a5, Col2a1, Col17a1, Col4a6, Col19a1,* Col9a3

Integrins: *Itga3, Itgb4*, Itga6*, Itgb5*

Laminins: *Lama5, Lamc2, Lama3, Lamb3*

MAPK family: *Mapk13, Dusp10, Map3k9, Mapkapk3*

RAS family: *Hras1, Fras1, Rab25, Rab27b, Diras2*

Kinases: *Ripk4, Limk2*

Other genes with relevance to epithelial or mammary development: *Odz2/Ten-M, Tac2, Ccnd2, Sostdc1, Pgr, Prom2, Jub, Msi2, Rbm35a, Tns4, Grb7, Trim29*, Tacstd1/Epcam**

B

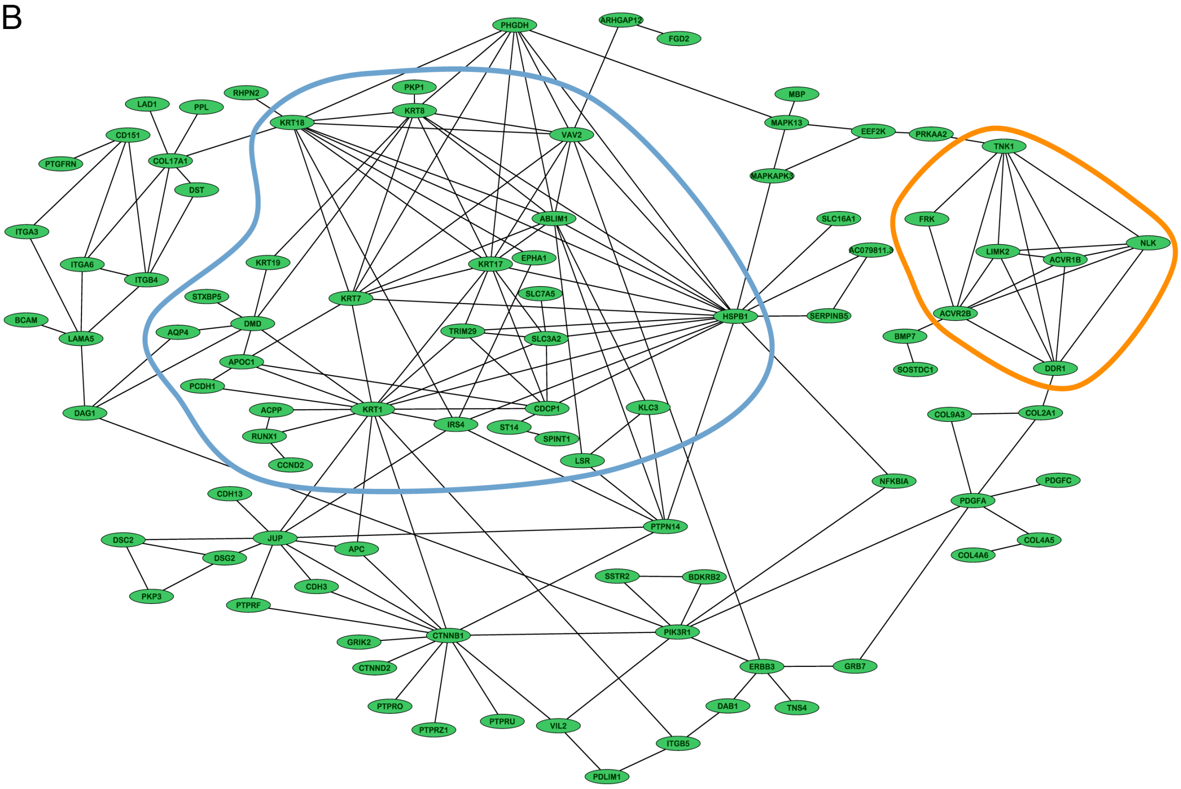

Transcriptomic characteristics of the mammary primordial epithelium.

(A) Select epithelial-enriched genes belonging to key developmental pathways. Genes or pathways that are known to be required for mammary primordial development are indicated in bold. Genes whose expression was validated as epithelial-enriched by immunohistochemistry, immunofluorescence, *in situ*

hybridisation, or qRT-PCR are indicated by an asterisk (*).

(B) A core network module comprised of 93 nodes was generated using human orthologues of physically interacting genes characteristic of E12.5 mammary primordial epithelium. Black lines represent protein-protein interactions. Network analysis of the primordial detects a large Keratin sub-module outlined in blue which connects with other highly interconnected nodes (or hubs) including JUP and CTNNB1. A small kinase-activin receptor sub-module is outlined in orange.
