# Supplementary material for: Transcriptome analysis of embryonic mammary cells reveals insights into mammary lineage establishment
Source: Breast Cancer Res. 2011 Aug 11;13(4):R79. doi: 10.1186/bcr2928 (PMC3236343; doi:10.1186/bcr2928)
Supplement: Additional file 5 — Transcriptomic characteristics of the mammary mesenchyme. (A) A figure summarising select genetic components of the mammary mesenchyme detected by array analysis. (B) A figure depicting network analysis of the mammary mesenchyme. [file bcr2928-S5.PDF]

A

Select mammary mesenchymal genes

Wnt pathway: *Wisp1, Fzd4, (Tcf4), Tcf15, Tcf19*
Notch pathway: *Dlk1*, Notch4**
Erbb pathway: *Hbegf, Tmeff2*
FGF pathway: *Fgf7*, Fgfr1*
TNF pathway: *Nfkbiz*
TGF pathway: *Tgfbi, Tgfbr2*, Tgfb1i1, Inhbb*
IGF pathway: *Igf1, Igfbp3*
Axon guidance: *Nrp2*, Nrp1, Robo4, Sema5a, Sema5b, Slit2, Ephb1, Epha3*
Homeobox-containing transcription factors: *Pitx2, Meox2, Lhx9, Hhex, Alx3, Zfhx4, Prrx2*
Transcription factors: *Dmrt2, Tbx18, Lmo2, Ldb2, Sox17, Sox18, Tbx15*, Gata6*, Klf12, Fxyd5, Tbx4, Tbx1, Klf4, Runx1t1, Klf6, Ets1*
Tight Junction components: *Cldn5*
Cadherins: *Cdh5, Pcdh12, Pcdh10, Cdh2, Pcdh19*
Collagens: *Col3a1, Col15a1 Col24a1 Col1a2 Col11a1 Col5a2 Col8a2 Col25a1*
Integrin signaling: *Ilk, Cav1**
Laminins: *Lama4*
RAS family: *Rasgrp3, Rasgrp2, Rassf2, Rab13, Ralb, Rhoc, Rab31*
Kinases: *Tek, Flt4, Camk2a, Flt1, Mylk, Prkg1, Nrk, Ror1, Camk2d, Pfkp, Ilk, Ror1*
Cell surface markers: *Cd93, Cd248, Cd38, Cd109,* CD248* (endosialin)
Chemokines: *Cxcl12, Cxcr4, Ccl11, Ccl12, Ccl21a*
Annexins: *Anxa5, Anxa6, Anxa3*
Adams: *Adamts1, Adamts9, Adam12, Adamts2, Adam19, Adamts4, Adamts3, Adam33*
Cell adhesion: *Icam2, Ncam2, Cdon, Ncam1*
Other genes of interest: *Emcn, Plek, Ogn, Dcn*,Vcam1, Esam, Angptl1, Csf1r, Fstl1, Ccna2, Rgs2*, Sparc**

B

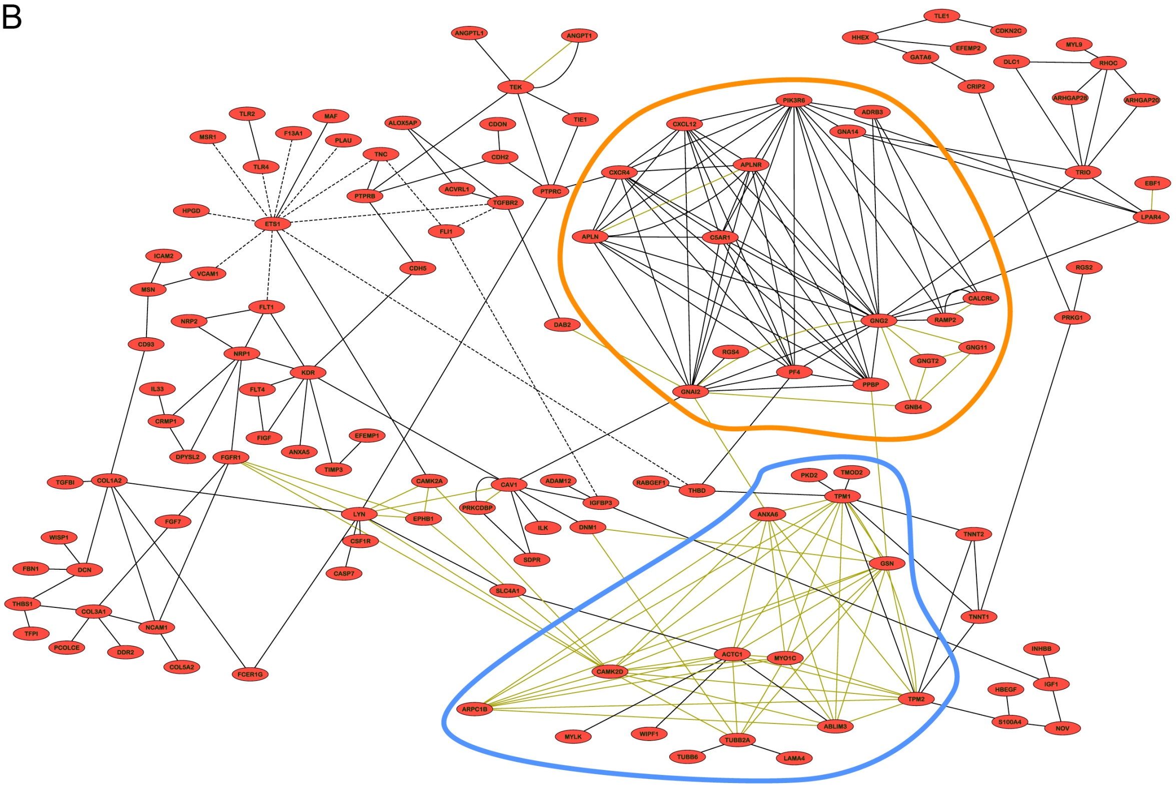

Transcriptomic characteristics of the mammary mesenchyme.

(A) Select mesenchymal-enriched genes belonging to key developmental pathways. Genes (or their markers) whose expression patterns validated as epithelial-enriched by immunohistochemistry, immunofluorescence, *in situ* hybridisation, or qRT-PCR are indicated by an asterisk (*).

(B) A core network module of E12.5 mammary primordial mesenchyme based on 125 nodes was generated using human orthologs of directly interacting genes characteristic of the of E12.5 mammary mesenchyme. Black lines represent protein-protein interactions. Nodes that have been shown to interact in complexes are connected together by yellow-green lines. Dotted lines represent transcriptional interactions. A sub-module comprised of several highly interconnected nodes representing G protein coupled receptor signalling and cytokines is outlined in orange. A cytoskeletal sub-module is outlined in blue and represents many signals that regulate cell integrity and architecture. Many of the mesenchymal network components are associated with angiogenesis and neural pathfinding and neural development consistent with the cell types known to be present within the mammary mesenchyme including vascular endothelial cells, pericytes and neural cells.
