# Supplementary material for: Transcriptome analysis of embryonic mammary cells reveals insights into mammary lineage establishment
Source: Breast Cancer Res. 2011 Aug 11;13(4):R79. doi: 10.1186/bcr2928 (PMC3236343; doi:10.1186/bcr2928)
Supplement: Additional file 9 — Prospective mediators of epithelial-mesenchymal interactions identified by network analysis. (A) A figure depicting the core interconnected network module. (B) A figure depicting how mesenchymal INHBB is poised to bind to epithelial ACVR1B and ACVR2B. [file bcr2928-S9.PDF]

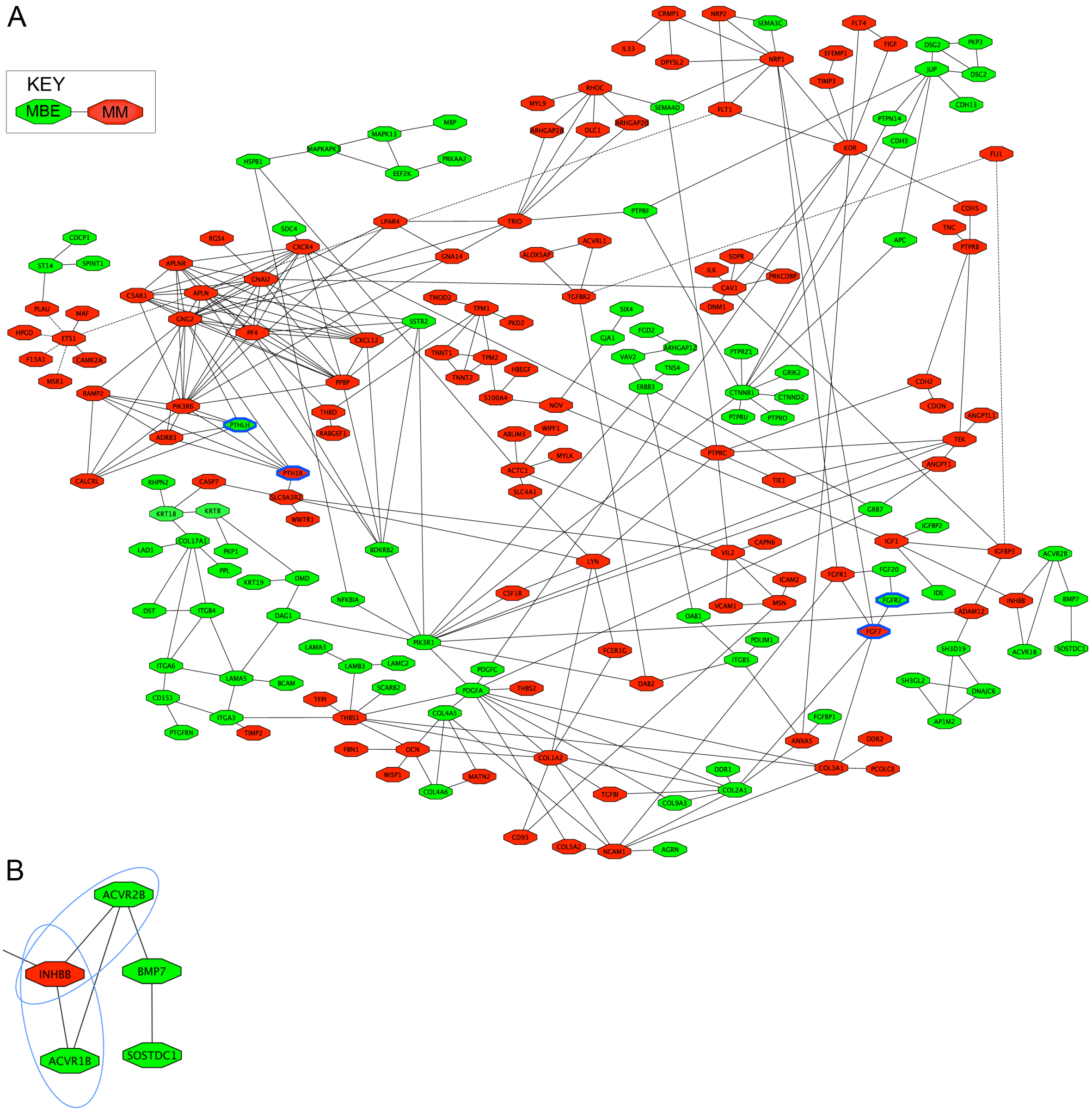

Prospective mediators of epithelial-mesenchymal interactions identified in the core interconnected network module.

(A) Core interconnected network module consisting of 197 nodes. Black lines represent protein-protein interactions. PTHLH and its cognate receptor PTHR; FGF7 and its cognate receptor, FGFR2 are outlined in blue. These nodes are known to interact in the mouse and were manually added or interconnected to assist in connecting the epithelial and mesenchymal genes obtained from the SAM analysis

comparing the two tissues with both Q<5% and local FDR<5%. Red represents mesenchymal nodes. Green represents epithelial nodes.

(B) Mesenchymal INHBB is poised to bind to epithelial ACVR1B and ACVR2B. Mesenchymal Inhibbin-B signalling to mouse mammary epithelial cells is required during puberty and pregnancy for normal mammary ductal elongation and alveolar development to occur. The interconnected nodes suggest that this tissue interaction also occurs during mammary primordial development.
