# Supplementary material for: Transcriptome analysis of embryonic mammary cells reveals insights into mammary lineage establishment
Source: Breast Cancer Res. 2011 Aug 11;13(4):R79. doi: 10.1186/bcr2928 (PMC3236343; doi:10.1186/bcr2928)
Supplement: Additional file 13 — Comparison of gene expression profiles of embryonic mammary cells with postnatal mammary cells. (A) Venn diagrams showing comparison of genes expressed by embryonic mammary primordial cells to those expressed by the cells contained within the terminal end bud and ductal microenvironments from [38]. (B) A heatmap showing the expression of a variety of key developmental, lineage, and progenitor markers in embryonic and postnatal mammary cell populations. (C) Venn diagram showing comparison of genes expressed by embryonic mammary primordial cells to those expressed by the conserved mouse/human MEC subpopulations and human fibroblasts from [13,55]. [file bcr2928-S13.PDF]

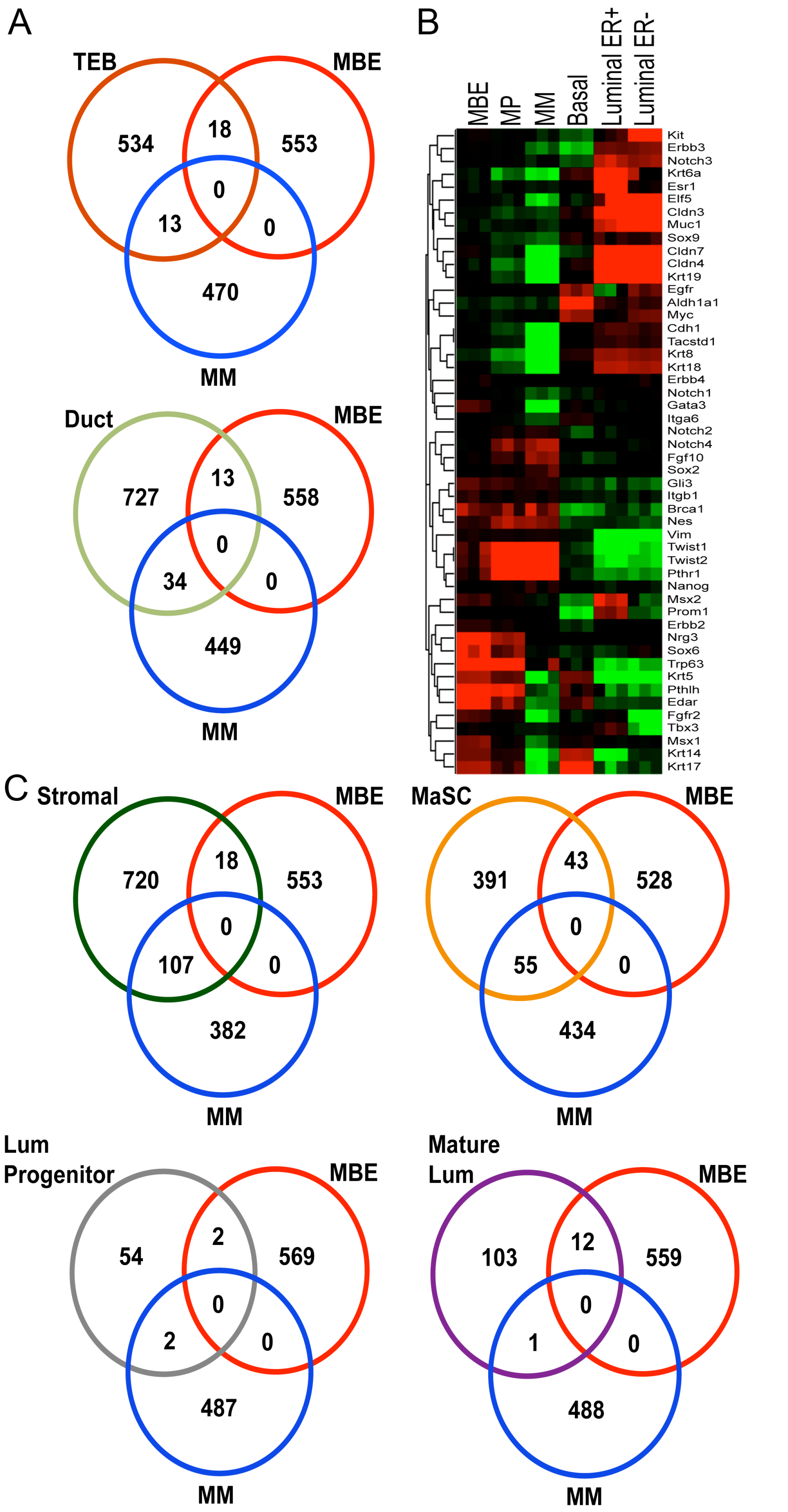

Comparison of gene expression profiles of embryonic mammary cells with postnatal mammary cells.

(A) Comparison of genes expressed by mammary primordial cells to those expressed by the postnatal mammary cell populations described by [38]. Venn diagrams showing the number of shared and unique genes expressed within mammary primordium compared to those expressed by the cells contained within the terminal end bud and ductal microenvironments, including adjacent stromal cells. Duct denotes ductal cells. TEB denotes terminal end bud. MBE denotes mammary bud epithelium. MM denotes mammary mesenchyme.

(B) Heatmap showing the expression of a variety of key developmental, lineage, and progenitor markers in primordial and postnatal mammary cell populations. Each horizontal line represents a probe set with red indicating high expression and green indicating low expression in the various tissue compartments.

(C) Comparison of genes expressed by mammary primordial cells to those expressed by the conserved mouse/human MEC subpopulations described by [13]. Venn diagrams showing the number of shared and unique genes expressed within mammary primordium compared to the three conserved mouse/human MEC subpopulations described by [13] and the stromal population described by [55]. MaSC denotes mammary stem cell, Lum Progenitor denotes luminal progenitor. Mature Lum denotes mature luminal. MBE denotes mammary bud epithelium. MM denotes mammary mesenchyme.
